# Leg length discrepancy, overgrowth, and associated risk factors after a pediatric tibial shaft fracture

**DOI:** 10.1186/s10195-021-00575-x

**Published:** 2021-03-15

**Authors:** Woo Young Choi, Moon Seok Park, Kyoung Min Lee, Kug Jin Choi, Hyon Soo Jung, Ki Hyuk Sung

**Affiliations:** Department of Orthopaedic Surgery, Seoul National University College of Medicine, Seoul National University Bundang Hospital, 82 Gumi-ro 173 Beon-gil, Bundang-Gu, Sungnam, 13620 Gyeonggi Korea

**Keywords:** Leg length discrepancy, Overgrowth, Pediatric, Tibial shaft fracture

## Abstract

**Background:**

This study was performed to investigate leg length discrepancy (LLD), overgrowth, and associated risk factors after pediatric tibial shaft fractures.

**Materials and methods:**

This study included 103 patients younger than 14 years of age (mean age 7.1 years; 75 boys, 28 girls) with unilateral tibial shaft fracture and a minimum follow-up of 24 months. LLD was calculated as the difference between the lengths of the injured and uninjured limbs. Overgrowth was calculated by adding the fracture site shortening from the LLD. Risk factors were assessed in patients with LLD < 1 cm and ≥ 1 cm and overgrowth < 1 cm and ≥ 1 cm.

**Results:**

Casting and titanium elastic nailing (TEN) were performed on 64 and 39 patients, respectively. The mean LLD and overgrowth were 5.6 and 6.4 mm, respectively. There were significant differences in sex (*p* = 0.018), age (*p* = 0.041), fibular involvement (*p* = 0.005), injury mechanism (*p* = 0.006), and treatment methods (*p* < 0.001) between patients with LLDs < 1 cm and ≥ 1 cm. There were significant differences in sex (*p* = 0.029), fibular involvement (*p* = 0.002), injury mechanism (*p* = 0.008), and treatment methods (*p* < 0.001) between patients with overgrowth < 1 cm and ≥ 1 cm. Sex and treatment methods were risk factors associated with LLD ≥ 1 cm and overgrowth ≥ 1 cm following pediatric tibial shaft fracture. The boys had a 7.4-fold higher risk of LLD ≥ 1 cm and 5.4-fold higher risk of overgrowth ≥ 1 cm than the girls. Patients who underwent TEN had a 4.3-fold higher risk of LLD ≥ 1 cm and 4.8-fold higher risk of overgrowth ≥ 1 cm than those treated by casting.

**Conclusions:**

Patients undergoing TEN showed greater LLD and overgrowth than those undergoing casting, with boys showing greater LLD and overgrowth than girls. Surgeons should consider the possibility of LLD and overgrowth after pediatric tibial shaft fractures, especially when performing TEN for boys.

**Level of evidence:**

Level III

## Introduction

Tibial shaft fractures account for about 1.1% of all pediatric fractures and are the second most common long bone fracture in a child, after forearm fractures [1, 2]. In addition, tibial and fibular fractures account for 21.5% of pediatric orthopedic trauma hospitalizations and are the second most frequent injury, after femoral fractures [3]. Also, for pediatric patients with polytrauma, tibia is the third most common fracture site after femur and humerus [4, 5].

A closed reduction followed by casting for pediatric tibial shaft fractures is the mainstay treatment method. Surgical treatment methods include external fixation, intramedullary nailing, crossed Kirschner wire fixation, and plate fixation. For intramedullary fixation, flexible intramedullary nails such as titanium elastic nails are mainly used rather than rigid intramedullary nails to prevent epiphyseal injuries of the proximal tibia. Treatment methods depend on the patient’s age, weight, fracture pattern, and the surgeon’s preference [6–12].

One of the common sequelae after a pediatric tibial shaft fracture is leg length discrepancy (LLD) owing to overgrowth of the injured tibia [13]. According to a number of studies that investigated the biomechanical effects of LLD, back pain, hip pain, and stress fractures have been reported as musculoskeletal disorders associated with LLD [14–16]. Most of the overgrowth has been shown to occur within 18 months after the fracture, and the average overgrowth at the time of follow-up was approximately 6 mm in cases of tibial shaft fractures [17]. This is thought to be due to the physiological processes associated with posttraumatic activation of the growth plate [18, 19].

Several studies have investigated the risk factors associated with overgrowth after pediatric femur shaft fracture, which included age, fracture site, fracture stability, and nail-to-canal diameter ratio [17, 20–26]. However, few studies have analyzed the risk factors for LLD and overgrowth after a tibial shaft fracture in children. Therefore, the aim of this study was to investigate LLD and overgrowth after a pediatric tibial shaft fracture and its associated risk factors.

## Materials and methods

The study protocol was approved by the institutional review board of our institution, and the requirement of informed consent from the participants was waived because of the retrospective nature of the study.

### Study population and data collection

We reviewed and retrieved the information of consecutive patients with a tibial shaft fracture between January 2003 and November 2018. Of these, patients with a unilateral tibial shaft fracture who were younger than 14 years of age and had a minimum follow-up of 24 months were included in this study. The exclusion criteria were as follows: (1) bilateral tibial shaft fracture, (2) ipsilateral or contralateral lower limb fractures, or (3) pathologic fracture due to congenital pseudoarthrosis, cerebral palsy, bone tumor, or osteogenesis imperfecta.

Through the examination of medical records, information such as sex, age, body mass index (BMI), follow-up duration, side of limb, injury mechanism, and treatment method was obtained. Injury mechanisms were divided into high-energy and low-energy injuries. Traffic accidents and falling from high places were defined as high-energy injuries, and slipping and falling from ground level was defined as low-energy injuries. All patients were treated by two pediatric orthopedic surgeons (MSP and KHS). Treatment methods included casting and titanium elastic nailing (TEN).

### Surgical procedure

All surgical procedures were performed under general anesthesia. The fractured limbs were cleaned and draped with the patient lying in the supine position. TEN was performed with antegrade methods after obtaining the appropriate reduction through closed manipulation under C-arm fluoroscopy. To prevent damage to the proximal physis of fractured tibia, medial and lateral proximal entry points were set at a distal position from the proximal epiphysis. A 2–3 cm longitudinal skin incision was made as a set entry point, and then cortical holes were made using an awl. Two titanium elastic nails with a diameter corresponding to the target of 80% of the total canal fill at the isthmus were adequately bent and inserted into the lateral and medial entry points. While confirming fracture reduction with C-arm fluoroscopy, the titanium elastic nails were advanced to the distal side of the fracture. Following confirmation of fracture reduction and positioning of the titanium elastic nails under fluoroscopic guidance, the tips of the titanium elastic nails were bent and cut away from the bone surface.

### Radiographic measurements

The radiographs of the patients were captured using a UT 2000 X-ray machine (Philips Research; Eindhoven, the Netherlands) according to our protocol, which is as follows: the anteroposterior (AP) and lateral radiographs included the tibia as a whole (from knee to ankle), while the beam was focused on the middle part. To assess limb length, AP standing long-cassette radiographs of the lower extremity (teleradiogram) were obtained by vertically entering the horizontal center beam to the patella height and vertical beam to the midline. The radiograph setting was 50 kVp and 5 mAs at a source-to-image distance of 200 cm with the patella facing forward. Until 2 years after the trauma, a teleradiogram was taken regularly once per year, because most of the overgrowths occur within 2 years after injury. Thereafter, if the LLD ≥ 1 cm, teleradiogram is recommended to be performed regularly once every 1–2 years. Otherwise, a regular examination was decided according to the parents’ need. All radiographic images were acquired digitally using a picture archiving and communication system (PACS, Infinitt; Seoul, South Korea), and radiographic measurements were performed using PACS software.

Fracture location and fracture stability were determined from the preoperative anteroposterior (AP) and lateral radiographs of the injured tibia. Fracture locations were classified as follows: proximal one-third, middle one-third, and distal one-third. Fractures were divided into length-stable and length-unstable fractures according to fracture stability. Transverse and short oblique fractures were classified as length-stable fractures; and long oblique, spiral, and comminuted fractures were classified as length-unstable fractures. Long oblique fractures were defined as when the angle between the fracture line and a line perpendicular to the long axis of the tibia was > 30°. From the postoperative AP and lateral radiographs of the tibia, fracture-site shortening was measured.

Whole limb length (WLL) was measured from the teleradiogram implemented at the time of the last follow-up, and the length from the top of the femoral head to the center of the tibial plafond was defined as WLL. LLD was measured as the injured limb length minus the uninjured limb length. Overgrowth was calculated by adding the fracture site shortening from the LLD. To investigate the risk factors for LLD and overgrowth, we divided our patients into those with LLD of < 1 cm or ≥ 1 cm, and those with an overgrowth of < 1 cm or ≥ 1 cm.

### Statistical methods

Descriptive statistical analysis was performed including the mean, standard deviation (SD), and proportion to summarize patient demographics. A comparison of variables between groups was performed using a Student’s *t*-test and the *χ*^2^ test. Multivariate logistic regression analysis was performed to identify the risk factors for an LLD ≥ 1 cm and overgrowth ≥ 1 cm in patients with a pediatric tibial shaft fracture. All statistical analyses were performed using SPSS software for Windows (Version 25.0; SPSS, Inc.; Chicago, IL, USA), and statistical significance was accepted when the *p*-values were < 0.05.

## Results

A total of 103 patients with a tibial shaft fracture were included in the analysis. The mean age of the patient at the time of fracture was 7.2 ± 3.3 years, and there were 75 (72.8%) boys and 28 (27.2%) girls.

There were 6 proximal one-third (5.8%), 40 middle one-third (38.8%), and 57 distal one-third (55.4%) fracture locations. In 24 patients (23.3%), the fracture was length stable; and in 79 patients (76.7%), it was length unstable. There were 66 high-energy (64.1%) and 37 low-energy (35.9%) injury mechanisms of fracture. The mean follow-up duration was 3.9 ± 2.0 years. Casting and TEN were performed on 64 (62.1%) and 39 (37.9%) patients, respectively, to treat tibial shaft fracture. The average fractured site shortening length that was observed after treatment was 0.8 ± 1.5 mm. The mean LLD and mean overgrowth were 5.6 ± 7.7 mm (95% confidence intervals [CI] 4.1–7.1) and 6.4 ± 7.6 mm (95% CI 4.9–7.9), respectively. Of the 103 patients, 24 (23.3%) had an LLD of ≥ 1 cm and 27 (26.2%) had an overgrowth of ≥ 1 cm (Table 1). Five patients (4.9%) had an LLD of more than 2 cm, and seven patients (6.0%) had an overgrowth of more than 2 cm. Three patients underwent epiphysiodesis, and one patient underwent tibial lengthening using an Ilizarov external fixator. One patient underwent hemiepiphysiodesis because of posttraumatic genu valgum.

**Table 1:** Summary of patient demographics

	Number of patients
Sex (male/female)	75/28
Age (years)	7.1 ± 3.3 (1.0–13.6)
Body mass index (kg/m^2^)	19.5 ± 4.3
Side of limbs (right/left)	53/50
Fracture location (proximal one-third/middle one-third/distal one-third)	6/40/57
Fibular involvement (yes/no)	43/60
Stability (length stable/length unstable)	24/79
Injury mechanism (high energy/low energy)	66/37
Follow-up duration (years)	3.9 ± 2.0 (2.0 to 13.0)
Treatment (cast/TEN)	64/39
Fracture site shortening (mm)	0.8 ± 1.5
LLD > 1 cm (yes/no)	24/79
Overgrowth > 1 cm (yes/no)	27/76

*TEN* titanium elastic nailing, *LLD* leg length discrepancy

There were significant differences in sex (*p* = 0.018), age (*p* = 0.041), fibular involvement (*p* = 0.005), injury mechanism (*p* = 0.006), and treatment methods (*p* < 0.001) between patients with LLD < 1 cm and those with LLD ≥ 1 cm. However, there were no significant differences in BMI, side of limbs, fracture location, fracture stability, and follow-up duration between the two groups (Table 2).

**Table 2:** Comparison of variables between patients with LLD < 1 cm and those with LLD ≥ 1 cm

	LLD < 1 cm (*N* = 79)	LLD ≥ 1 cm (*N* = 24)	*p*-value
Sex (male/female)	53/26	22/2	0.018
Age (years)	6.8 ± 3.6	8.0 ± 2.0	0.041
Body mass index (kg/m^2^)	19.4 ± 3.7	20.3 ± 5.6	0.380
Side of limbs (right/left)	40/39	13/11	0.762
Fracture location (proximal one-third/middle one-third/distal one-third)	6/26/47	0/14/10	0.051
Fibular involvement (yes/no)	27/52	16/8	0.005
Stability (length stable/length unstable)	17/62	7/17	0.438
Injury mechanism (high energy/low energy)	45/34	21/3	0.006
Follow-up duration (years)	3.9 ± 2.1	4.1 ± 1.7	0.670
Treatment (cast/TEN)	57/22	7/17	< 0.001

*LLD* leg length discrepancy, *TEN* titanium elastic nailing

There were significant differences in sex (*p* = 0.029), fibular involvement (*p* = 0.002), injury mechanism (*p* = 0.008), and treatment methods (*p* < 0.001) between patients with an overgrowth < 1 cm and those with an overgrowth ≥ 1 cm. However, there were no significant differences in age, BMI, side of limbs, fracture location, fracture stability, and follow-up duration between the two groups (Table 3).

**Table 3:** Comparison of variables between patients with overgrowth < 1 cm and those with overgrowth ≥ 1 cm

	Overgrowth < 1 cm (*N* = 76)	Overgrowth ≥ 1 cm (*N* = 27)	*p*-value
Sex (male/female)	51/25	24/3	0.029
Age (years)	6.8 ± 3.6	7.9 ± 2.3	0.052
Body mass index (kg/m^2^)	19.3 ± 3.7	19.8 ± 5.5	0.687
Side of limbs (right/left)	37/39	16/11	0.345
Fracture location (proximal one-third/middle one-third/distal one-third)	6/25/45	0/15/12	0.062
Fibular involvement (yes/no)	25/51	18/9	0.002
Stability (length stable/length unstable)	17/59	7/20	0.707
Injury mechanism (high energy/low energy)	43/33	23/4	0.008
Follow-up duration (years)	3.9 ± 2.1	4.0 ± 1.7	0.904
Treatment (cast/TEN)	56/20	8/19	< 0.001

*TEN* titanium elastic nailing

There were significant differences in age (*p* = 0.041), fibular involvement (*p* < 0.001), and injury mechanism (*p* < 0.001) between cast and TEN treated patients. However, there were no significant differences in sex, BMI, side of limbs, fracture location, fracture stability, and follow-up duration between the two groups (Table 4).

**Table 4:** Comparison of variables between patients treated by cast and TEN

	Cast (*N* = 64)	TEN (*N* = 39)	*p*-value
Sex (male/female)	46/18	29/10	0.783
Age (years)	6.8 ± 3.6	8.0 ± 2.0	0.041
Body mass index (kg/m^2^)	19.4 ± 3.7	20.3 ± 5.6	0.380
Side of limbs (right/left)	31/33	22/17	0.432
Fracture location (proximal one-third/middle one-third/distal one-third)	6/21/37	0/19/20	0.066
Fibular involvement (yes/no)	15/49	28/11	< 0.001
Stability (length stable/length unstable)	13/51	11/28	0.358
Injury mechanism (high energy/low energy)	30/34	36/3	< 0.001
Follow-up duration (years)	3.9 ± 2.1	4.1 ± 1.7	0.670
LLD > 1 cm (yes/no)	7/57	17/22	< 0.001
Overgrowth > 1 cm (yes/no)	8/56	19/20	< 0.001

*TEN* titanium elastic nailing, *LLD* leg length discrepancy

Multivariate logistic regression analysis showed that sex (*p* = 0.014) and treatment methods (*p* = 0.011) were risk factors associated with an LLD ≥ 1 cm after pediatric tibial shaft fracture. The boys had a 7.4-fold higher risk of LLD ≥ 1 cm than the girls. Children who underwent TEN had a 4.3-fold higher risk of LLD ≥ 1 cm than those who underwent casting. However, other variables, including age, fracture location, fibular involvement, and injury mechanism, were not associated with LLD ≥ 1 cm after pediatric tibial shaft fracture (Table 5).

**Table 5:** Risk factors for leg length discrepancy (≥ 1 cm) after pediatric tibial shaft fracture

Variables	*B*	Exp (B)	95% CI for Exp (B)	*p*-value
Sex	2.003	7.410	1.499–36.620	0.014
Age (per year)	−0.157	0.855	0.686–1.033	0.164
Fracture location				
Proximal one-third	−18.195	0.000	0.000	0.999
Middle one-third	0.941	0.278	0.075 to 1.027	0.102
Distal one-third (reference)				
Fibular involvement	0.924	2.521	0.775–8.201	0.125
Injury mechanism high energy versus low energy	1.261	3.529	0.834–14.939	0.087
Treatment Cast versus TEN	1.468	4.341	1.394–13.516	0.011

*CI* confidence interval, *TEN* titanium elastic nailing

In addition, sex (*p* = 0.030) and treatment methods (*p* < 0.001) were risk factors associated with an overgrowth ≥ 1 cm after pediatric tibial shaft fracture. The boys had a 5.4-fold higher risk of overgrowth ≥ 1 cm than the girls. Patients who underwent TEN had a 4.7-fold higher risk of overgrowth ≥ 1 cm than those who underwent casting. However, other variables, including age, fracture location, fibular involvement, and injury mechanism, were not associated with overgrowth ≥ 1 cm after pediatric tibial shaft fracture (Table 6).

**Table 6:** Risk factors for overgrowth (≥ 1 cm) after pediatric tibial shaft fracture

Variables	*B*	Exp (B)	95% CI for Exp (B)	*p*-value
Sex	1.689	5.413	1.321–22.185	0.019
Age (per year)	−0.150	0.861	0.699–1.060	0.258
Fracture location				
Proximal one-third	−18.676	0.000	0.000	0.999
Middle one-third	0.787	2.197	0.751–6.426	0.211
Distal one-third (reference)				
Fibular involvement	0.978	2.660	0.872–8.117	0.086
Injury mechanism high energy versus low energy	0.962	2.618	0.678–10.102	0.136
Treatment cast versus TEN	1.560	4.760	1.578–14.356	0.006

*CI* confidence interval, *TEN* titanium elastic nailing

## Discussion

In general, LLD has been accepted as a result of overgrowth of the fractured tibia or femur [13]. If LLD at skeletal maturity exceeds or is expected to exceed 2–2.5 cm, length equalization procedures such as epiphysiodesis or limb lengthening should be considered [27]. Therefore, it is clinically important to identify risk factors for LLD and overgrowth after a pediatric tibial shaft fracture. This study demonstrated that LLD and overgrowth after pediatric tibial shaft fractures was significantly associated with sex and treatment methods. The patients treated by TEN had greater LLD and overgrowth than those treated with casting, with the boys exhibiting greater LLD and overgrowth than the girls.

A number of studies have reported overgrowth and its risk factors after femur shaft fracture in children [17, 22–24]. They found that length-unstable fracture, low nail-to-canal diameter ratio, and younger age were associated with overgrowth after a pediatric femur shaft fracture.

There have only been two studies investigating LLD and overgrowth after a pediatric tibial shaft fracture. Stilli et al. assessed overgrowth in children who had received conservative treatment for femur and tibial shaft fractures [17] and found a greater amount of overgrowth in younger patients than in patients 5 years after conservative treatment. In addition, they showed that a greater amount of overgrowth was found in cases of diaphyseal fractures with a greater initial displacement, with significant angular deviation and overriding of bone fragments. Lee et al. assessed LLD in 27 patients with flexible intramedullary nail fixation for femur and tibial shaft fractures [13] and showed that a younger age at the time of injury was significantly associated with LLD.

In this study, sex and treatment methods were a significant factor for LLD ≥ 1 and overgrowth ≥ 1 cm after pediatric tibial shaft fracture. The boys had a 7.4-fold higher risk of having an LLD of ≥ 1 cm and a 5.4-fold higher risk of having an overgrowth of ≥ 1 cm than the girls. Moreover, the five patients who had an LLD of > 2 cm and seven patients who had an overgrowth of > 2 cm were all boys. It is tempting to postulate that hyperemia results in growth stimulation, which has a greater effect on the relatively dormant growth plate of boys [28]. In addition, high-energy injuries are more frequent in boys who are relatively active compared with girls, and eventually, surgical treatment such as TEN may become more frequent than treatment with cast. This could be the reason for the high risk of LLD and overgrowth in boys. However, our study showed that boys had a greater LLD (3.0 mm) and overgrowth (2.6 mm) compared with girls, and this difference does not appear to be a clinically significant problem.

Patients treated with TEN had a 4.3-fold higher risk of having an LLD of ≥ 1 cm and a 4.7-fold higher risk of having an overgrowth ≥ 1 cm than those treated with casting. Among the five patients with an LLD of > 2 cm, four were initially treated with TEN and one with casting. Among the seven patients with an overgrowth of > 2 cm, six were initially treated with TEN and one with casting. Halanski et al. conducted an animal study to investigate the association between periosteum disruption and overgrowth after long bone fractures [29]. They analyzed the growth rates after performing procedures to disrupt periosteal fibers on the tibia of skeletally immature rabbits and showed that growth acceleration occurred at both the proximal and distal tibial growth plates after the periosteal procedures. Generally, surgical treatment with TEN is performed rather than conservative treatment with casting when treating displaced pediatric tibial shaft fractures. The displacement of the fracture site may disrupt the periosteum; therefore, it may be reasonable for LLD and overgrowth to be observed in patients treated with TEN rather than with casting.

Canavese et al. reported that there was no significant difference in the results between nonoperative management and operative treatment with elastic stable intramedullary nailing for patients with immature skeleton who had displaced tibial fractures but no associated fibula fractures [30]. Marengo et al. demonstrated that the use of elastic stable intramedullary nailing for displaced tibial shaft fractures in children and adolescents aged ≥ 11 years and weighing ≥ 50 kg did not lead to worse outcomes [31]. Furthermore, our study results showed that age, BMI, and presence of fibular involvement were not risk factors for LLD and overgrowth in patients with pediatric tibial shaft fracture.

There were limitations to this study. First, the minimum follow-up duration of our cohort was 24 months, which may affect the study outcomes. However, there was no significant difference in follow-up duration between patients with LLD < 1 cm and ≥ 1 cm, and between patients with overgrowth < 1 cm and ≥ 1 cm. In addition, our analysis showed that follow-up duration was not associated with LLD and overgrowth after pediatric tibial shaft fractures. Further long-term follow-up studies are required. Second, this study included TEN as a surgical treatment method and did not include other fixation methods, such as external fixation and plating. Our study showed that LLD and overgrowth are more likely to occur when patients are treated with TEN compared with when patients are treated with casting, although lesser complications have been reported for TEN than for other techniques (plate fixation, external fixation) [32, 33]. Further study including patients treated using other surgical techniques is needed. Third, the study population between patients treated with casting and TEN is not homogeneous (64 versus 39) due to the retrospective nature of this study, which might be a bias affecting the study results. To control the bias, we performed multivariate analysis for identifying the risk factors. Therefore, we think that our study may provide clinically meaningful results. Fourth, this study included patients aged < 14 years; thus, the age range was wide. The degree of overgrowth may vary depending on the residual potential of growth from the time of injury. However, our multivariate analysis showed that age was not associated with the risk of LLD or overgrowth occurring after tibial shaft fractures in pediatric cases. A further study with a narrow range of age groups is needed.

In conclusion, the patients who underwent TEN showed greater LLD and overgrowth than those who underwent casting, with boys exhibiting greater LLD and overgrowth than girls. However, it is difficult to say whether the results have clinical significance because the differences in LLD and overgrowth were not significant between sexes and treatment methods. Nevertheless, because the parents of pediatric patients find it difficult to accept even a slight LLD or overgrowth as a result of the fracture, surgeons need to consider the possibility of LLD and overgrowth in pediatric tibial shaft fracture cases, especially when performing TEN for boys.

## Data Availability

The dataset supporting the conclusion of this article is available on request to the corresponding author.

## Abbreviations

LLD: Leg length discrepancy
TEN: Titanium elastic nailing
BMI: Body mass index
AP: Anteroposterior
WLL: Whole limb length
CI: Confidence intervals
